# Individual assessment of ventilatory acclimatization at high altitude

**DOI:** 10.1113/EP091920

**Published:** 2024-04-18

**Authors:** Erik R. Swenson, Martin Burtscher

**Affiliations:** ^1^ Department of Medicine Geisel School of Medicine at Dartmouth Lebanon New Hampshire USA; ^2^ Department of Sport Science University of Innsbruck Innsbruck Austria

**Keywords:** acclimatization, altitude, alveolar, blood gases, mountain sickness, pulmonary ventilation

1

Acclimatization to high altitude is essential for the prevention of high‐altitude illnesses such as acute mountain sickness (AMS), or life‐threatening high‐altitude cerebral and pulmonary oedemas, but also for the partial restoration of the initial loss of (submaximal) exercise performance during acute exposure to high altitude. Acclimatization represents a complex process including several physiological and molecular mechanisms of different time courses (Mallet et al., 2023). Improvement of blood oxygenation and oxygen delivery to tissues constitute the primary goal of acclimatization, which is achieved to a large extent by increased pulmonary ventilation (ventilatory acclimatization to hypoxia, VAH). In this issue of *Experimental Physiology*, Isakovich et al. present an intriguing approach on how to assess the individual VAH status during high‐altitude ascent (Isakovich et al., 2024).

While VAH is almost completed within an about 8‐day sojourn to altitudes between 4000 and 4500 m, more than 2 months may be necessary when ascending to extreme altitudes, that is, above 8000 m (West, 1988). When sea‐level or low‐altitude residents ascend to high altitude, pulmonary ventilation progressively increases with hypoxic stimulation of peripheral chemoreceptors (in the carotid bodies) preventing a greater fall of blood oxygen content and associated severe hypoxemia. According to the alveolar gas equation, hyperventilation is paralleled by an increase in the alveolar partial pressure of oxygen ($P_{AO_{2}}$) and a related decrease in the alveolar partial pressure of carbon dioxide ($P_{\mathrm{AC}O_{2}}$).

The O_2_–CO_2_ diagram shows the combined changes in $P_{AO_{2}}$ and $P_{\mathrm{AC}O_{2}}$ levels measured at various altitudes, which differ characteristically between non‐acclimatized and acclimatized individuals, indicating lower $P_{\mathrm{AC}O_{2}}$ values for the same $P_{AO_{2}}$ values in more acclimatized subjects (Rahn & Otis, 1949). This in turn means that higher $P_{AO_{2}}$ (and arterial $P_{O_{2}}$, $P_{aO_{2}}$) levels can be maintained by hyperventilation in acclimatized individuals despite lower $P_{\mathrm{AC}O_{2}}$ (and related arterial $P_{CO_{2}}$, $P_{\mathrm{aC}O_{2}}$) levels. This may be a consequence of sustained stimulation of central chemoreceptors (on the ventral brain stem medullary surface) related to renal bicarbonate loss with acclimatization and reduction in the bicarbonate concentration of the cerebrospinal fluid, but also changes in cerebral blood flow may alter chemoreceptor stimulation. Notably, above a certain altitude (of about 7000 m) $P_{AO_{2}}$ is almost kept constant at about 35 mmHg whereas $P_{\mathrm{AC}O_{2}}$ steeply decreases to levels between 10 and 15 mmHg on the Everest summit (8848 m) (Grocott et al., 2009; West, 1988). Accordingly, the extent of hyperventilation caused by decreasing O_2_ levels with increasing altitude and the related changes in alveolar and blood gases, may all provide certain information on the acclimatization status and the associated risk of developing high‐altitude illnesses. While using a single and easy‐to‐measure parameter such as peripheral oxygen saturation ($S_{pO_{2}}$) for the evaluation of VAH and the individual AMS risk led to somewhat contradictory results, it seems reasonable to assume that the recording of two (or more) parameters may be more promising, that is, $S_{pO_{2}}$ and the end‐tidal $P_{CO_{2}}$ ($P_{\mathrm{ETC}O_{2}}$, the level of CO_2_ present at the end of an exhaled breath), which are easily accessible under field conditions by the use of pulse oximetry and capnometry.

Isakovich et al. introduce such field measurements for the evaluation of individual VAH (Isakovich et al., 2024). The authors repeatedly recorded $S_{pO_{2}}$ and $P_{\mathrm{ETC}O_{2}}$ values from 46 study participants during a 10‐day ascent to 5160 m. They performed area under the curve (AUC) calculations on individual modified Fenn diagrams ($P_{\mathrm{ETC}O_{2}}$ plotted against $S_{pO_{2}}$) and formed three groups representing the smallest, medium and largest magnitudes of AUC, corresponding to a high, medium and low degree of VAH. In addition, these recordings were also taken from a separate group with the same ascent profile but taking acetazolamide (125 mg twice a day), which is known to promote acclimatization (Swenson, 2014). Surprisingly, highly acclimatized participants with smaller AUCs on modified Fenn diagrams did not show different AMS scores compared to those with lower levels of acclimatization. This finding challenges the clinical relevance of drawing Fenn diagrams when climbing high altitudes. On the other hand, this may simply be a consequence of the generally low AMS scores reported during the slow ascent (Isakovich et al., 2024) and/or the differences in individual susceptibility to AMS which may have blurred the association between the level of acclimatization and AMS development. This point would have been better established if each participant could have served as his or her own control but necessitating an impractical two ascents separated by enough time to return ventilatory control back to low altitude levels. Although it cannot be assumed with certainty that AUCs on individual Fenn diagrams enable a clear distinction between inter‐individual acclimatization status, some confirmation may be derived from the fact that the use of acetazolamide resulted in lower AUC values. From a physiological perspective, Isakovich et al. (2024) present stimulating data on the assessment of individual acclimatization status, that is, VAH, by the use of a novel measurement method that is feasibly applied during trekking and high‐altitude climbing (at least up to about 5500 m). However, future studies will need to be performed to validate its clinical and practical relevance.

## AUTHOR CONTRIBUTIONS

Martin Burtscher wrote the first draft and Erik Swenson provided extensive revision of the manuscript. Both authors contributed equally to the writing. Both authors have read and approved the final version of this manuscript and agree to be accountable for all aspects of the work in ensuring that questions related to the accuracy or integrity of any part of the work are appropriately investigated and resolved. All persons designated as authors qualify for authorship, and all those who qualify for authorship are listed.

## CONFLICT OF INTEREST

The authors declare no conflict of interest

## FUNDING INFORMATION

No funding to be reported.
